# Heterogeneity of pathological prion protein accumulation in the brain of moose (*Alces alces*) from Norway, Sweden and Finland with chronic wasting disease

**DOI:** 10.1186/s13567-023-01208-3

**Published:** 2023-09-08

**Authors:** Diego Sola, Linh Tran, Jørn Våge, Knut Madslien, Tram T. Vuong, Sirkka Liisa Korpenfelt, Erik O. Ågren, Gustav Averhed, Maria Nöremark, Kaisa Sörén, Mats Isaksson, Cristina Acín, Juan José Badiola, Dolores Gavier-Widén, Sylvie L. Benestad

**Affiliations:** 1 Centro de Encefalopatías Y Enfermedades Transmisibles Emergentes, Universidad de Zaragoza, IA2, IIS Aragón, 50013 Zaragoza, Spain; 2 National Veterinary Institute (SVA), 75189 Uppsala, Sweden; 3 Department of Biomedical Sciences and Veterinary Public Health, Swedish University of Agricultural Sciences (SLU), Box 7028, 75007 Uppsala, Sweden; 4 WOAH Reference Laboratory for CWD (SLB), Norwegian Veterinary Institute (NVI), Postboks 64, 1431 Ås, Ås Norway; 5 Finnish Food Authority, Animal Health Diagnostic Unit, 00790 Helsinki, Finland

**Keywords:** CWD, moose, prion disease, chronic wasting disease, prions, transmissible spongiform encephalopathy, Norway, Finland, Sweden

## Abstract

Prion diseases are a group of neurodegenerative, transmissible, and fatal disorders that affect several animal species. They are characterized by the conformational conversion of the cellular prion protein (PrP^C^) into the pathological prion protein (PrP^Sc^). In 2016, chronic wasting disease (CWD) gained great importance at European level due to the first disease detection in a wild reindeer (*Rangifer tarandus*) in Norway. The subsequent intensive CWD surveillance launched in cervids resulted in the detection of CWD in moose (*Alces alces*), with 11 cases in Norway, 3 in Finland and 4 in Sweden. These moose cases differ considerably from CWD cases in North American and reindeer in Norway, as PrP^Sc^ was detectable in the brain but not in lymphoid tissues. These facts suggest the occurrence of a new type of CWD. Here, we show some immunohistochemical features that are clearly different from CWD cases in North American and Norwegian reindeer. Further, the different types of PrP^Sc^ deposits found among moose demonstrate strong variations between the cases, supporting the postulation that these cases could carry multiple strains of CWD.

**Supplementary Information:**

The online version contains supplementary material available at 10.1186/s13567-023-01208-3.

## Introduction

Transmissible spongiform encephalopathies (TSEs) or prion diseases are progressive, fatal, neurodegenerative diseases caused by transformed host proteins called prions (proteinaceous infectious particles). Prions are formed by misfolding of the normal host cellular prion protein (PrP^C^) into the disease-associated isoform, PrP^Sc^ or PrP^res^, “res” denoting resistance to proteinase K treatment. Prions accumulate as fibrillar aggregates in the central nervous system, causing neurodegenerative changes and ultimately leading to death. Prions are considered to be devoid of nucleic acids and are able to cause infection in susceptible hosts upon natural or experimental transmission [1].

TSEs affect both animals and humans. In animals, the TSE archetypes are scrapie in sheep and goats, bovine spongiform encephalopathy (BSE) in cattle and chronic wasting disease (CWD) in cervids, and in humans, Creutzfeldt-Jakob disease (CJD), Gerstmann-Sträussler-Scheinker disease (GSS), and fatal familial insomnia (FFI).

There are different TSE types which differ in relation to the origin of the prions as well as natural routes of transmission. TSEs which are contagious in field conditions such as classical scrapie and CWD spread between animals, either by direct contact with infected animals or by indirect infection from prion contaminated environment; contagious rates can be high [2]. Accumulation of prions in peripheral tissues is a common feature of contagious and environmentally transmissible TSEs and may occur after foetal-maternal transmission [3–5]. Sporadic TSEs are generally considered to be of spontaneous origin. In animals, Nor98, also called atypical scrapie, was first recognised in old sheep in Norway in 1998 [6] and was later described to occur sporadically in most European countries [7–9], USA, Australia and New-Zealand [10, 11]. Subsequently, atypical BSE was detected in old cattle [12], with two different presentations according to the electrophoretic migration of their PrP^res^ in SDS-PAGE gels, the low or L-type (L-BSE) with lower position than classical BSE, and the high or H-type (H-BSE), with higher position than classical BSE. L-BSE was detected for the first time in Italy [13] while H-BSE was first described in France [14]. Nor98/atypical scrapie, as well as L-type and H-type BSE are considered to arise spontaneously in old animals [6–15] and the release of prions into the environment does not appear to reach the necessary levels for horizontal transmission [16] and thus contagion.

CWD was first identified as a neurodegenerative disease in a captive mule deer (*Odocoileus hemionus*) in Colorado, USA, in 1967 and was formally diagnosed as a disease with neuropathological features characteristic of prion diseases in 1978 [17]. Since then, CWD has been reported in free-ranging and captive cervids in North America, affecting also white-tailed deer (*Odocoileus virginianus*), Rocky Mountain elk (*Cervus elaphus nelson*i), wapiti (*Cervus canadensis*), moose (*Alces alces*) and red deer (*Cervus elaphus elaphus*). Today, CWD occurs with increasing prevalence and geographical range in North America in cervid populations [18–21]. The disease has also been detected in South Korea [22] after the unfortunate import of infected subclinical CWD-infected cervids, and was described for the first time in Europe in a wild reindeer (*Rangifer tarandus*) in Norway in 2016 [23]. This was also the first description of CWD in reindeer and the biochemical and immunohistochemical (IHC) features were similar to those in CWD isolates from North America [23]. The detection of CWD in Norway led to the implementation of an extensive/intensified CWD surveillance programme in Norway [24] and other Northern European countries [25, 26]. CWD surveillance of moose in Norway, Finland, and Sweden has been described [25]. Since 2005, more than 36 200 moose have been tested in Norway, of which 11 tested positive for CWD. In Sweden, since 2007, more than 1700 moose have been tested for CWD and four positive cases have been detected to date (June 2023). Finland has tested more than 800 moose since 2006, detecting two positive CWD cases [25], and detected a third case in moose since this publication.

An initial molecular and immunohistochemical characterisation of the three first CWD cases in moose in Norway [27], showed differences from CWD in North America [28], as well as from reindeer in Norway [27]. To characterise Norwegian CWD prions, strain typing studies after experimental transmission and adaptation to bank voles (*Myodes glareolus*) were conducted and results were compared with North American CWD strains. Based on differences in the incubation times, deposition of prions in the brain, neuropathological and biochemical characteristics in the bank voles, it was concluded that Norwegian CWD prion strains were distinct from the North American strains, indicating that the latter were not likely to be the direct cause of the detected cases of CWD in Norway. Furthermore, it was shown that CWD in reindeer and moose in Norway were caused by different strains, supporting that circulation of CWD between species in Norway was unlikely, unless adaptation has taken place. Biochemical differences were also observed among the three first CWD cases in moose in Norway, which were reflected in the isolation of two different bank vole adapted strains. [29]. These results were corroborated in transmission studies into transgenic and gene targeted mice expressing deer or elk PrP, which confirmed differences from North American CWD and between Norwegian moose and reindeer CWD [30], and differences among the moose isolates [29, 30]. Interestingly, adaptation of one Norwegian isolate during iterative passages in these mice resulted in prions with biological properties that are characteristic of North American strains.

In the present study, cases of moose CWD from Norway, Sweden, and Finland were characterized by IHC, applying a panel of monoclonal antibodies (mAbs). The aim of this work was to obtain a better understanding of the neuropathological presentation and PrP^Sc^ deposition in the brain of naturally occurring CWD in moose in the Nordic countries and to identify any similarities and variations between cases.

## Materials and methods

### Animals and tissues

#### Description of the animals

The moose investigated were CWD positive cases detected by the surveillance programmes in Norway, Sweden, and Finland. Country of origin, location, age and sex are reported in Tranulis et al. [25], except for an additional moose from Norway. In summary, 13 animals were investigated, seven from Norway (No); one male and six females, ranging from age 12 to 20 years, four from Sweden (Sw); all females, ranging from age > 10 to 16 years, and two from Finland (Fi); both females, aged 15 and 18 years. The animals were designated as No1 to No7, Sw1 to Sw4, Fi1 and Fi2. Sequencing of the moose prion protein gene (*PRNP*) revealed polymorphism at codon 109. All moose were KK109, except No4 and No6 which were QQ109.

#### Description of the samples and tests

*Primary diagnostic analyses of the spoon sample:* Samples of brain stem at the level of the obex (hereafter referred to as “obex”) and of retropharyngeal lymph nodes were collected as part of the surveillance programme. The screening diagnostic tests (TeSeE ELISA; Bio-Rad Laboratories, Inc., Hercules, CA, USA) and/or HerdCheck ELISA; (IDEXX Laboratories, Westbrook, USA) for detection of protease-resistant core of PrP^Sc^ (PrP^res^) were applied. The CWD diagnosis was confirmed by western blot testing (TeSeE WESTERN BLOT; Bio-Rad Laboratories, Inc., Hercules, CA, USA). Following positive results, the whole brain, and additional lymph nodes and tonsils, if available, were collected. The brain was divided mid-sagittally. One half was fixed in 10% neutral buffered formalin, and the other half was frozen at −20 °C. The lymph nodes were equally divided, and formalin fixed or frozen.

*Brain sampling for ELISA:* To investigate PrP^Sc^ distribution in the brain by ELISA, samples from 16 different brain areas were obtained from the unfixed brain from CWD positive moose in Sweden and Norway (Additional file 1). The ELISA OD values of individual brain areas were grouped into seven major brain areas: frontal cortex, temporal cortex, occipital cortex, thalamus, mesencephalon, obex and cerebellum. All cases detected in Norway could be assessed in all seven brain areas, while all cases detected in Sweden, except Sw4, could be assessed in all selected brain areas. In the case of the moose detected in Finland, it was not possible to assess the different selected areas by ELISA.

### Analyses of the additional brain samples

#### ELISA

Most of the moose brain areas were analysed only by the TeSeE™ SAP combi kit from Bio-Rad, ELISA test, and thereafter also by HerdChek* BSE-Scrapie ELISA test from IDEXX. The screening diagnostic tests (TeSeE™ SAP combi kit; Bio-Rad Laboratories, Inc., Hercules, CA, USA) and/or HerdChek* BSE-Scrapie ELISA test (IDEXX Laboratories, Westbrook, USA) for detection of protease-resistant core of PrP^Sc^ (PrP^res^) were applied. All the samples were analysed at the Norwegian National Veterinary Institute (NVI), one of the four WOAH (founded OIE) reference laboratories for CWD.

TeSeE™ SAP Combi ELISA was performed following the manufacturer’s instructions. Briefly, 250 µL of the homogenate sample was incubated for 10 min at 37 °C with 250 µL of denaturing solution, proteinase K solution (proteinase K in buffer A). The digestion was stopped by addition of 250 µL of clarifying solution, buffer B. After centrifugation at 20 000 × *g* for 10 min, the pellet was denatured in 25 µL buffer C for 5 min at 100 °C then diluted with 125 µL reagent R6. 100 µL was added into the ELISA plate and incubated 30 min at 37 °C. The plate was washed, incubated with respectively conjugate solution (30 min at 5 °C), and substrate (30 min in darkness at room temperature). Stop solution was added to the plate and OD values were read at 450 nm and 620 nm.

For the HerdChek ELISA test, following the manufacturer’s instructions, 120 µL of homogenate was mixed with 30 μL diluent solution (D1 and D2), and 100 μL of the mixture was loaded on to the antigen-capture plate for 45 min at room temperature with shaking. After washing, the plate was incubated with respectively conditioning buffer CB (10 min), conjugate anti-PrP antibody CC (45 min), TMB (15 min in darkness). Stop solution was added to the plate and OD values were read at 450 nm and 620 nm.

#### Immunohistochemistry protocol

Brain, lymph nodes, and tonsil samples were formalin-fixed for > 48 h and processed by standard histopathological techniques. IHC was used to visualize PrP^Sc^ distribution. Briefly, tissue sections on poly-L-lysine glass slides were deparaffinised, rehydrated, treated in 98% formic acid for 30 min, and autoclaved for antigen retrieval at 121 °C for 30 min in 0.01 M citric acid pH 6.0. Endogenous peroxidase activity was inhibited with blocking reagent (EnVisionTM + System HRP [(AEC)] DAKO, Glostrup, Denmark) for 10 min.

Non-specific antigenic sites were blocked with 5% bovine serum albumin (BSA) in Tris-buffered saline (TBS). The sections were incubated overnight at 4 °C with one of the monoclonal mAbs 12B2 (1:5000), 9A2 (1:4000), L42 (1:2000), SAF 84 (1:10 000) and P4 (1:3000). Several antibodies with different epitopes (sheep prion protein [PrP] numbering) were used for IHC: SAF84 (aa 167–173) were obtained from Bertin Pharma (Montigny-le-Bretonneux, France), L42 (aa 148–153) from R-Biopharm (Darmstadt, Germany), 9A2 (aa 102–104) and 12B2 (aa 93–97) from Wageningen Bioveterinary Research (Lelystad, Netherlands) and RIDA mAB P4 (aa 84–104) from R-Biopham (Germany).

Sections were then incubated for 45 min at 37 °C with secondary antibody, and the chromogen AEC (EnVisionTM + System HRP [(AEC)] DAKO, Glostrup, Denmark) was used for 10 to 15 min to visualize the immunostaining. Finally, the sections were counterstained with haematoxylin solution and mounted.

In each run, tissues from CWD-negative and CWD-positive moose and reindeer were added as negative and positive controls.

#### Immunohistochemistry scoring

The deposition of prions in tissues as detected by IHC is referred to as PrP^Sc^. Regarding the pattern of PrP^Sc^ deposition, the classification made by Jeffrey’s group [31, 32] was applied, in which they establish different types of deposition. Of these, the following types were observed in this study: fine punctate, coarse granular, intraneuronal, intraglial, linear, perineuronal and stellate. For simplification, the 16 areas were grouped into seven main brain areas as illustrated in Additional file 1 in red lines. The scoring system assesses the intensity and extent of distribution of PrP^Sc^ accumulation applying the following scale: 0 no stain; 1 mild; 2 moderate; 3 striking. For each animal, the amount of each “PrP^Sc^ type” was calculated as the average of the scores given in the seven brain areas. The amount of total PrP^Sc^ was calculated as the average of the scores of the seven PrP^Sc^ types.

Histopathological assessment of vacuolar degenerative changes in haematoxylin–eosin-stained sections was attempted but could not be evaluated due to artefactual vacuolation caused by autolysis.

## Results

### Neuroanatomical distribution and immunolabelling profiles of PrP^Sc^ in CWD-affected moose

The immunohistochemical patterns observed in CWD-positive moose by using five different antibodies were investigated and compared with the pattern obtained from a positive wild reindeer from Norway. This positive control showed an immunohistochemical pattern similar to that described in North American CWD [28]. In the negative controls, no immunostaining was observed in both moose and reindeer (Additional file 2). In none of the moose, PrP^Sc^ deposition was detected in the lymphoid tissues.

**Figure 1:**
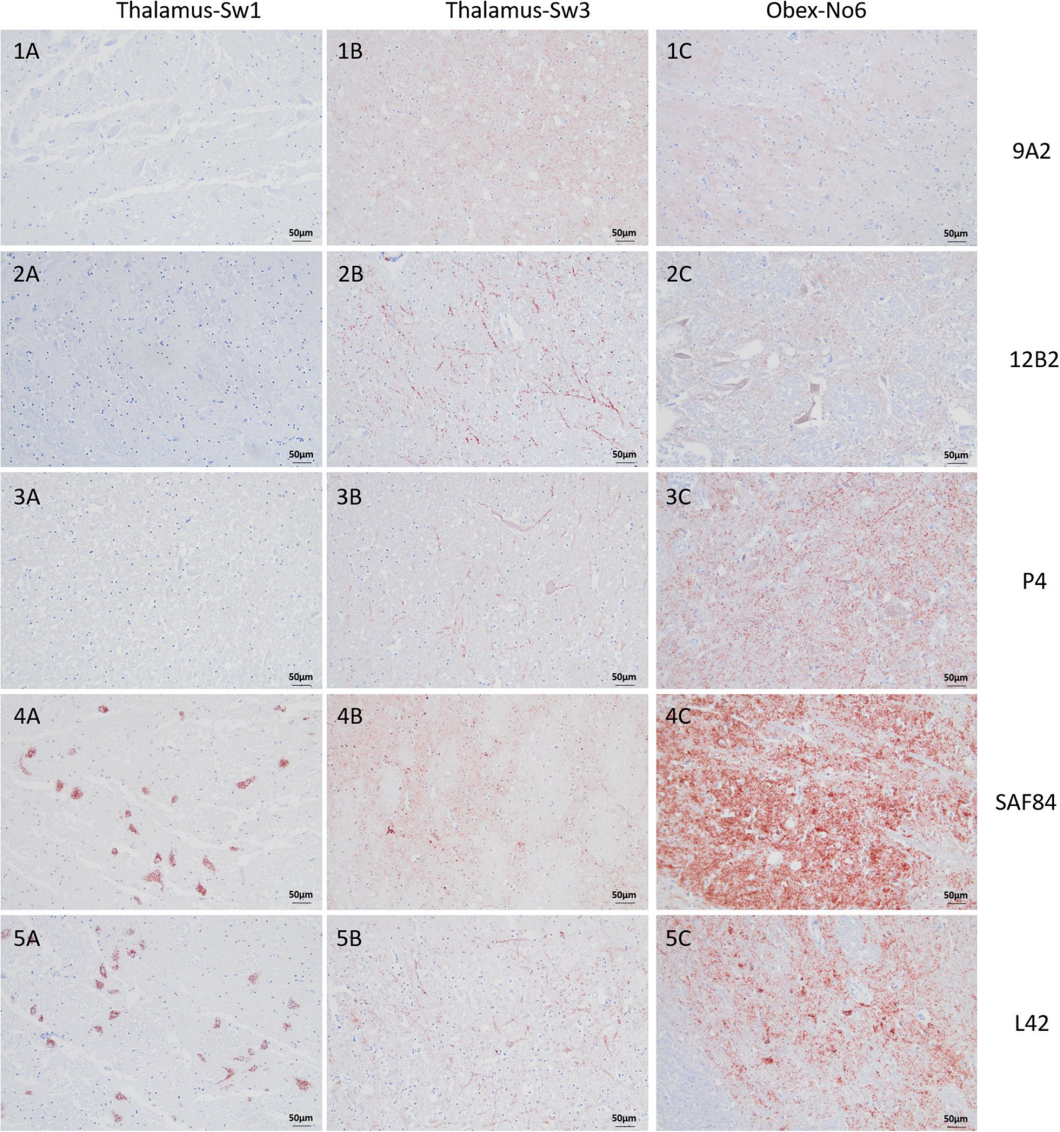
**Differences in PrP**^**Sc**^** immunolabelling in the thalamus and obex of CWD-affected moose with five different anti-PrP antibodies.** Immunohistochemistry was performed on brain sections of CWD-positive Swedish (Sw) moose (cases number 1 and 3) and Norwegian (No) moose (case number 6). The antibodies used are indicated on the right side of the figure. mAb 9A2, 12B2 and P4 have epitopes directed against PrP N-terminus, while mAb SAF84 and L42 have central epitopes. All sections were counterstained with haematoxylin. (Bar = 50 μm).

An overview of the PrP^Sc^ deposition patterns obtained with each of the five anti-PrP antibodies used is given in Figure 1. L42 and SAF84 antibodies clearly detected PrP^Sc^ in the brain of all animals. With N-terminal antibodies 12B2, 9A2, and P4, most of the moose were negative (as shown for Sw1), except Sw3 and No6.

**Figure 2:**
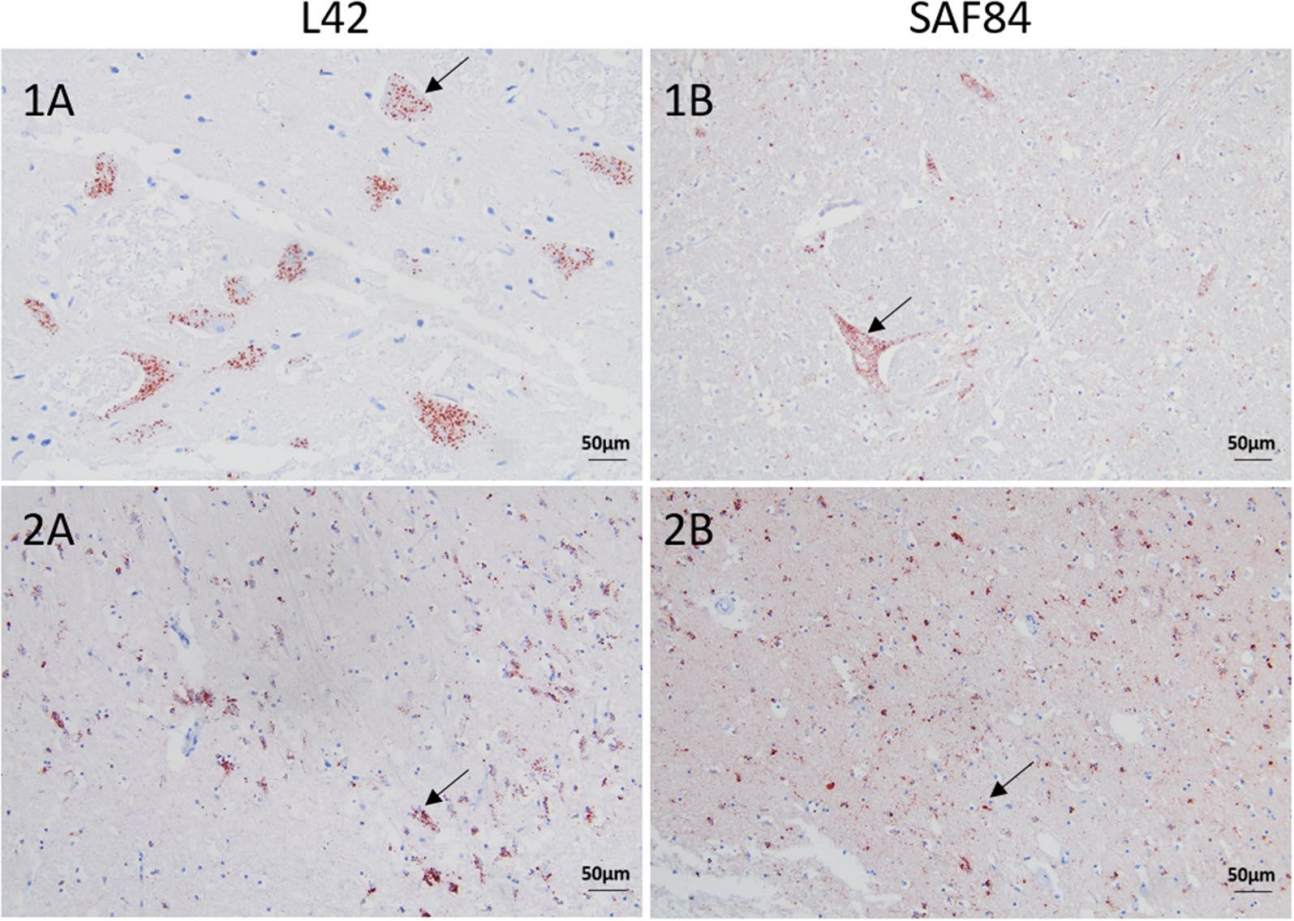
**Intracellular PrP**^**Sc**^** accumulation in brain sections of moose with CWD.** Immunohistochemistry was performed with anti-PrP mAbs L42 and SAF84, as indicated, with moose No1. Note the intraneuronal and intraglial staining at the level of the obex (panels 1A-1B) and frontal cortex (panels 2A-2B), respectively, as indicated by arrows. All sections were counterstained with haematoxylin. (Bar = 50 μm).

**Figure 3:**
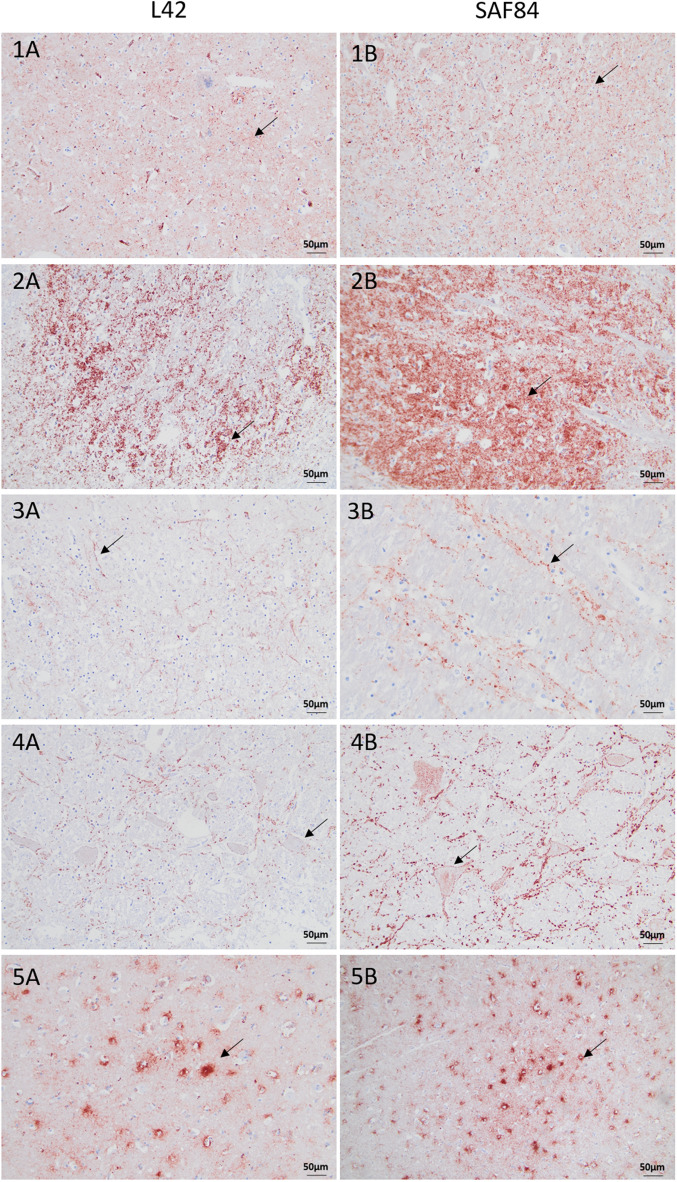
**Extracellular PrP**^**Sc**^** accumulation in brain sections of moose with CWD.** Immunohistochemistry was performed with anti-PrP mAbs L42 and SAF84, as indicated. Note the fine punctuate staining at the level of the thalamus with moose No1 (panel 1A) and No3 (panel 1B), the coarse granular PrP^Sc^ staining at the level of the obex with moose No6 (panels 2A-2B), the linear PrP^Sc^ at the level of the obex with moose No1 (panel 3A) and No3 (panel 3B), the perineuronal PrP^Sc^ staining at the level of the obex found only in moose Sw3 (4A-B), and the stellate PrP^Sc^ staining in the frontal cortex found only in moose No6 (panels 5A-5B). All sections were counterstained with haematoxylin. (Bar = 50 μm).

PrP^Sc^ deposits were observed both intracellularly and extracellularly with the core antibodies L42 and SAF84 (Figures 2 and 3, respectively).

**Figure 4:**
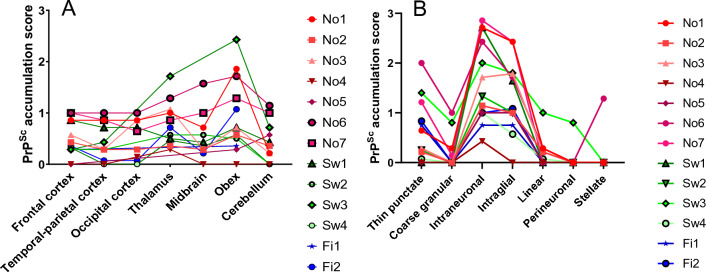
**Neuroanatomical distribution and profiles of PrP**^**Sc**^** in CWD-affected moose.** **A** PrP^Sc^ accumulation score in different brain areas of Norwegian (No), Swedish (Sw) and Finnish (Fi) CWD-affected moose. **B** PrP^Sc^ profiles of the moose. SAF84 mAbs was used for quantification.

Graphic representations of PrP^Sc^ accumulation score as a function of the brain area or the profile of deposition are shown as Figures 4A and B for all moose. A great heterogeneity among animals was observed. To facilitate visualisation and showing details of single cases, cases are shown and discussed by country of detection below.

#### Swedish cases

**Figure 5:**
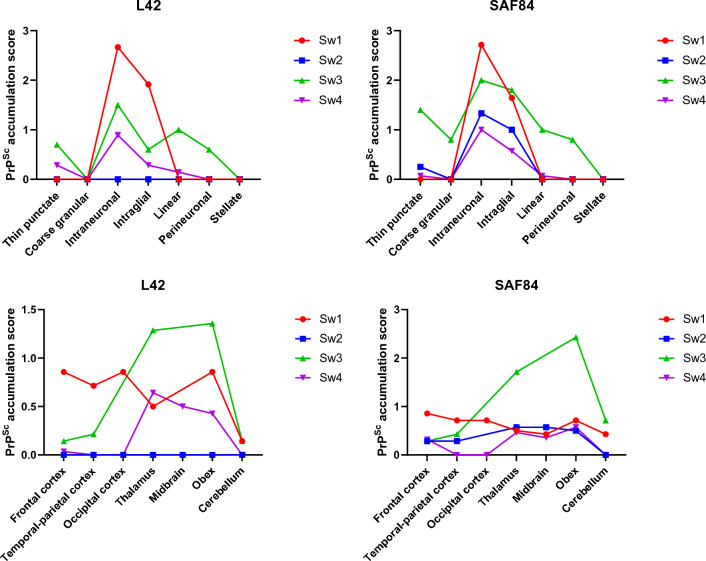
**Neuroanatomical distribution and profiles of PrP**^**Sc**^ **in Swedish CWD-affected moose with L42 and SAF84 mAbs.**

**Figure 6:**
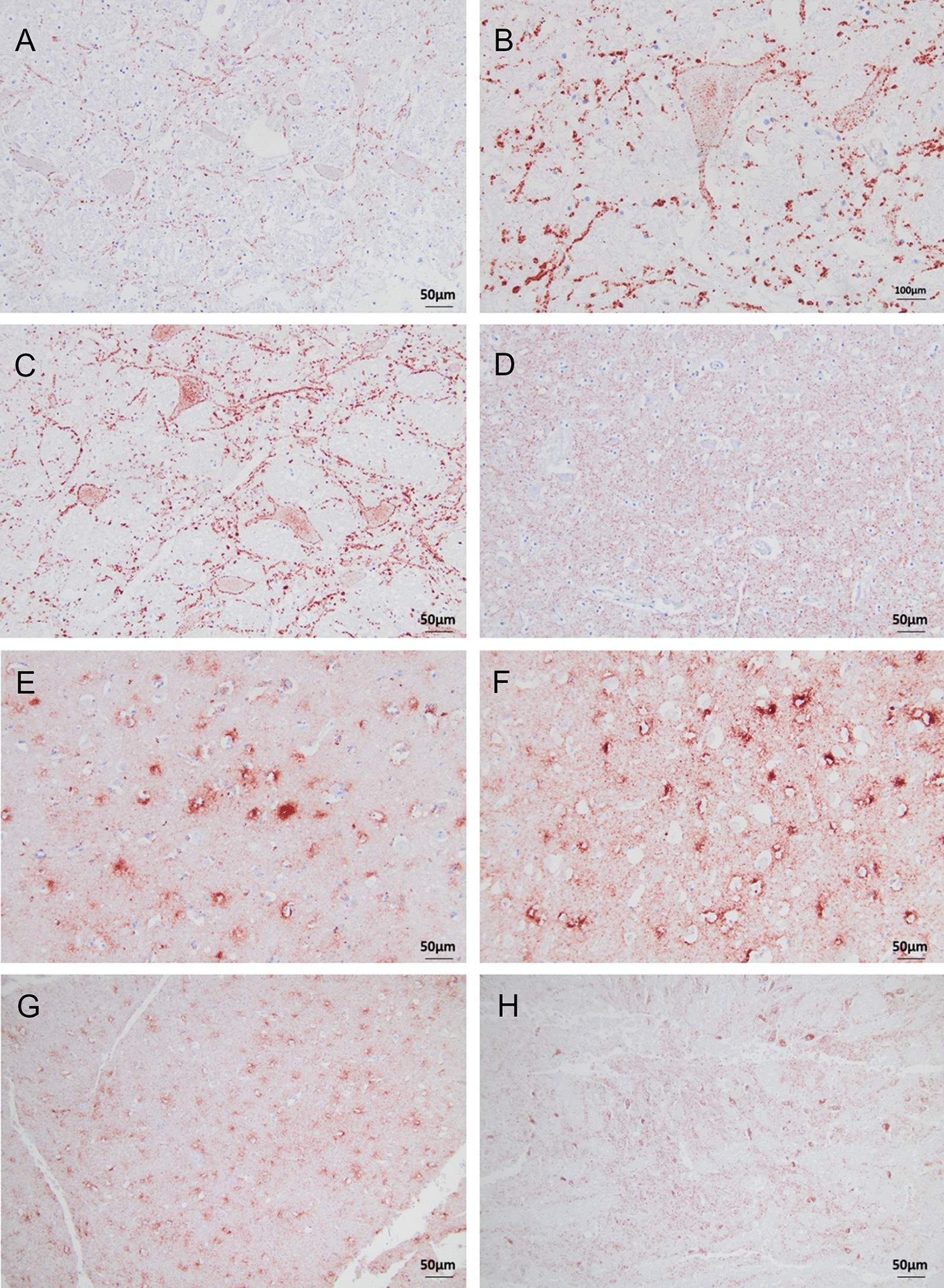
**PrP**^**Sc**^** immunolabelling in CWD-affected moose Sw3 and No6 with different anti-PrP antibodies.** **A-C** Obex in CWD-affected moose Sw3 with SAF84 (**A**) (Bar = 50 μm), L42 (Bar = 100 μm) (**B**) and 12B2 (Bar = 50 μm) (**C**), showing linear and perineural or both linear and perineural PrP^Sc^ staining, respectively (Bar = 50 μm). **D** Thalamus of Sw3 with 12B2 showing fine punctate PrP^Sc^ staining (Bar = 50 μm). **E–G** Cerebral cortex of CWD-affected moose No6 with SAF84 (Bar = 50 μm) (**E**), L42 (Bar = 50 μm) (**F**) and 12B2 (Bar = 50 μm) (**G**) showing stellate PrP^Sc^ staining (Bar = 50 μm) (**H**) Midbrain of CWD-affected moose No6 with L42 showing fine punctate PrP^Sc^ staining (Bar = 50 μm). All sections were counterstained with haematoxylin.

All Swedish cases had high levels of intraneuronal and intraglial PrP^Sc^ deposition. Significantly lower was the PrP^Sc^ fine punctate pattern as it was only observed in Sw2 at the level of the thalamus and in Sw3 at the level of the thalamus and obex. Moose Sw3 appeared clearly different from the other cases as it showed a much more intense pattern of fine punctate and coarse granular staining in the neuropil and also additional patterns not observed in the other moose, such as the linear and the perineuronal patterns, which were detected mainly in the thalamus, obex and in the frontal brain at the level of the caudate nucleus (Figures 5 and 6).

PrP^Sc^ deposition in the different brain areas varied among cases and also with different antibodies. For example, in Sw1, the frontal, temporal, parietal and occipital cortex were the main sites of deposition, with low levels in the cerebellum, with L42 mAbs. Sw3 showed the highest scores in the obex with both L42 and SAF84 mAbs and in Sw2 PrP^Sc^ was not detected with L42 mAbs (Figure 5).

#### Norwegian cases

**Figure 7:**
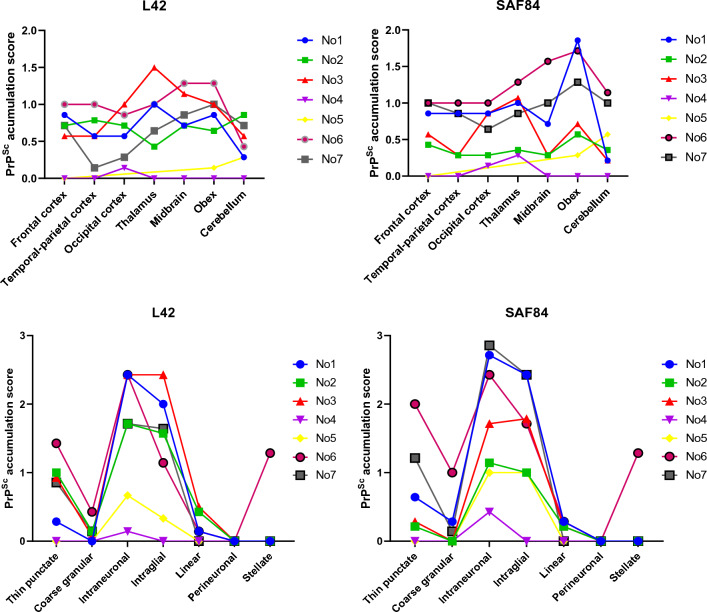
**Neuroanatomical distribution and profiles of PrP**^**Sc**^** in Norwegian CWD-affected moose with L42 and SAF84 mAbs.**

The Norwegian moose showed a similar immunohistochemical PrP^Sc^ deposition profile, mostly dominated by the intraneuronal and intraglial patterns, followed by the fine punctate pattern (Figure 7). However, one moose (No6) also showed a stellate deposition pattern, present throughout the cerebral cortex, which was not observed in the other moose (Figure 6).

PrP^Sc^ anatomical distribution showed intensity variations among Norwegian moose, but most brains showed PrP^Sc^ deposits in frontal cortex, parietal cortex, occipital cortex, thalamus, midbrain, medulla, and cerebellum. No4 only showed faint deposition at thalamic and occipital cortex level (Figure 7).

**Figure 8:**
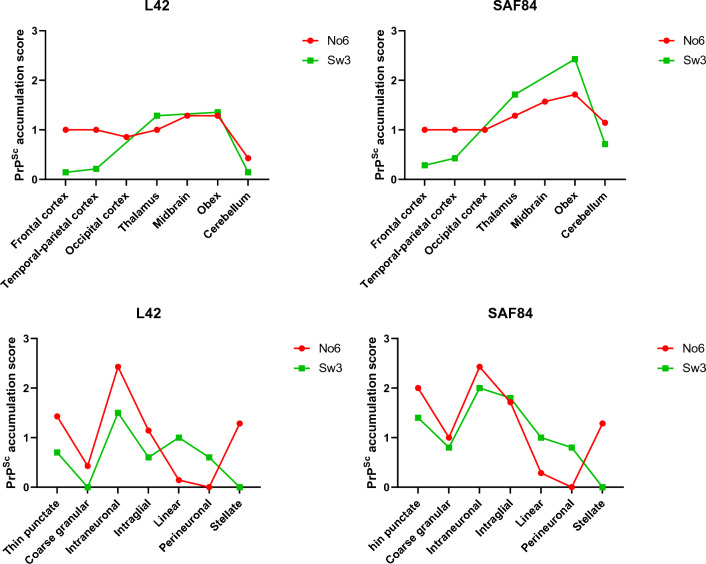
**Comparison of the neuroanatomical distribution and profiles of PrP**^**Sc**^** in Norwegian CWD-affected moose (No6) versus Swedish CWD-affected moose (Sw3) with L42 and SAF84 mAbs.**

Since one Swedish moose (Sw3) and one Norwegian moose (No6) showed some differences in the PrP^Sc^ patterns as compared with the other Swedish and Norwegian moose, their PrP^Sc^ patterns and brain distribution, with two antibodies, L42 and SAF84, were graphically compared to visualise any possible similarities between these two animals (Figure 8). The diagrams illustrated some similarities in both PrP^Sc^ patterns and distribution but also clear differences.

#### Finnish cases

**Figure 9:**
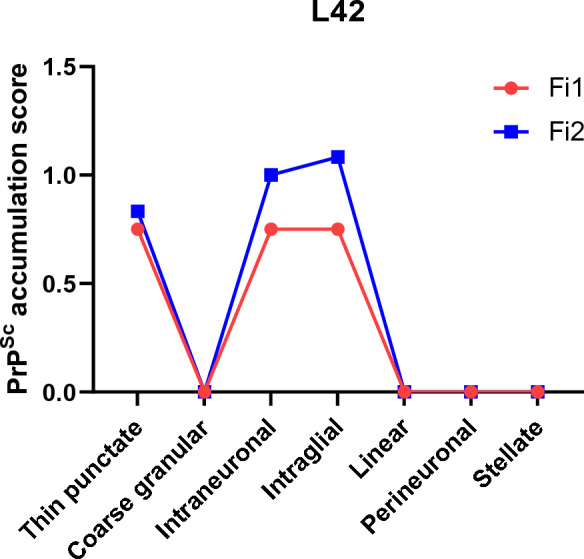
**PrP**^**Sc**^** profiles in the brain of Finnish moose with CWD.** The PrP^Sc^ profiles were obtained with L42 antibody.

The two cases detected in Finland showed similar deposition patterns. Fine punctate, intraneuronal, and intraglial PrP^Sc^ predominated. Fi2 showed PrP^Sc^ in all brain areas except for the cerebellum, whereas in Fi1 only frontal cortex and medulla were available, both showing PrP^Sc^ (Figure 9).

#### Total PrP^Sc^ comparison

**Figure 10:**
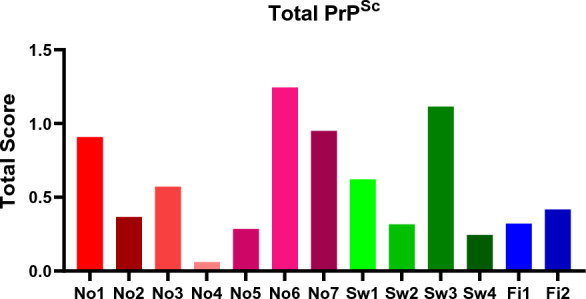
**Total PrP**^**Sc**^** content in the brains of in Swedish (Sw), Norwegian (No), and Finnish (Fi) moose with CWD.** The scale bar signifies the total PrP^Sc^ score found in the animals from 0 to 3. It represents the mean intensity of all deposit types found in all animals.

The magnitude of total PrP^Sc^ (all patterns combined) immunolabelling in the different moose is shown in Figure 10. Large variations in total PrP^Sc^ accumulation were observed amongst moose. No1, No6, No7, and Sw3 animals stand out with the highest PrP^Sc^ accumulation (Figure 10) (Table 1).

**Table 1:** **Identification of the moose investigated.**

ID	Country	Age (years)	Sex	Clinical observations
No1: 16-P138	Norway	13	Female	Abnormal behaviour, killed
No2: 16-P153	Norway	14	Female	Found dead with trauma
No3:17-CD11399	Norway	13	Female	Hunted but with abnormal behaviour (post-mortem detected hip dislocation)
No4: 18-CD24724	Norway	15	Female	Abnormal behaviour, killed (post-mortem detected hip dislocation and ethmoid tumour)
No5: 19-CD14225	Norway	20	Female	Hunted, no clinical signs
No6: 19-CD24854	Norway	12	Female	Found dead
No7: 21-CD41	Norway	13	Male	Killed due to a broken leg
Sw1: 19-VLT000541	Sweden	16	Female	Emaciated, walking in circles, seemed blind
Sw2: 19-VLT000876	Sweden	16	Female	Emaciated, behavioural changes
Sw3: 19-VLT002322	Sweden	> 10	Female	Hunted. Clinically healthy (but altered behaviour)
Sw4: 20-VLT002459	Sweden	14	Female	Lame, indolent
Fi1: 1256/20	Finland	15	Female	Found dead
Fi2: 2054/20	Finland	18	Female	Emaciated, in lying position

### Neuroanatomical distribution of PrP^Sc^ in CWD moose brains by ELISA

The immunohistochemical results showed above were consistent with the results of the rapid ELISA tests, which showed positivity in each of the brain areas and each moose, but strong variations between individuals. High OD-values were found in the frontal cortex, thalamus, midbrain, and obex, followed by the rest of the brain areas. The less-affected areas were the cerebellum and the occipital cortex (Table 2). For the Finnish moose the ELISA results were not available for the different brain areas.

**Table 2:**
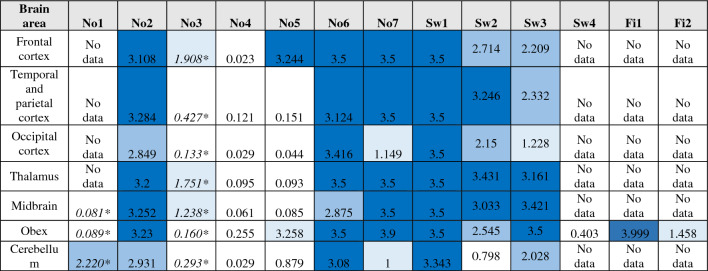
**OD values in the IDEXX ELISA test applied to different brain areas.**

^*^ Data obtained by Bio-Rad test are indicated in the table, as no material was available for testing with the IDEXX test, which normally gives higher OD values in the moose. The intensity of blue colours (from lighter to darker) represents a higher score detected in the ELISA test.

## Discussion

The emergence of new prion strains is phenotypically visible when a propagated prion does not maintain the same biochemical and pathogenic characteristics as the original strain. This phenomenon can occur both during interspecies and intraspecies transmission [33]. In addition to these, different strains can arise spontaneously or through spontaneous mutation in the prion protein gene, *PRNP*, encoding PrP^C^, making it more susceptible to misfolding [34, 35]. With the discovery of the prion protein and its association with the disease, diagnostic techniques for the characterisation of prion strains such as immunohistochemistry were developed [32–37].

It is very important to understand how the different strains of CWD detected in Europe behave biologically in European moose and to understand the epidemiology of the disease in this species to be able to inform surveillance and management strategies in different cervid species in relation to different strains. This study applied immunohistochemical phenotyping to cases of moose CWD identified in the Nordic countries, to better characterise these cases neuropathologically and understand the biology of the prions involved.

In this immunohistochemical study, similarities but also differences in the phenotypical presentations were found amongst moose, suggesting a possible involvement of different strains of CWD. Importantly, these differences were present regardless of the country of origin. Previous studies suggest the presence of different strains of CWD in North America [38]. The conformational selection model [39] interprets strains as mixtures or “clouds” of different PrP^Sc^ conformations, among which there is a predominant one that determines the phenotypic characteristics of the disease. However, in this “cloud”, minority PrP^Sc^ isoforms, sometimes called “sub-strains” or “quasi-species”, may be present, which are transmitted and replicate together with the dominant conformer below the detection threshold of current diagnostic techniques. The immunohistochemical findings indicate phenotypic heterogeneity among the cases but cannot provide information about possible presence of sub-strains or mixture of strains. To investigate the type of prion strain(s) in Norwegian CWD cases, the biochemical characteristics of disease-associated PrP and transmission to rodent models were conducted and provided preliminary evidence of several prion “strains” [29, 30]. Based on preliminary observations of CWD in moose in Norway, which described the intraneuronal and intraglial PrP^Sc^ deposits [27], together with their old age, and especially the absence of detectable PrP^Sc^ in lymphoid tissues using traditional diagnostic methods (ELISA HerdChek from IDEXX and TeSeE from Bio-Rad), WB (TeSeE WESTERN BLOT from Bio-Rad) and immunohistochemistry, it was postulated that these features were associated to a sporadic form of CWD [27]. The more extensive investigation in this study confirmed the initial findings and also revealed additional pattern presentations, namely, fine punctate deposition throughout the neuropil in several moose, marked stellate deposition in No6, and marked linear as well as perineuronal pattern in Sw3. The stellate and perineuronal patterns were only observed in a single animal.

IHC was performed with a panel of five anti-PrP mAbs generated against different parts of PrP. In early studies of CWD in Norwegian moose, a lack of staining with mAbs that bind to the N-terminal tail of PrP such as the 12B2 and 9A2 antibodies was observed [27]. This indicates that in these cases, this part of the protein had been degraded by endogenous proteases. These findings were corroborated for all moose in this study, except of Sw3 and No6. In these two animals, PrP^Sc^ was detected at the thalamic level with antibodies 12B2, 9A2, and P4, suggesting variability in the N-terminal proteinase K-cleavage sites.

All the moose analysed were at least 10 years old or older, which implies that affected animals were all in old age. There are rare reports of male moose reaching 21 years of age and female moose reaching 25 years of age [40], but most moose are harvested at earlier age. Management and hunting cause different age distribution between males and females moose populations. While female moose can reach old age, bulls rarely turn over 10 years old. The difference in age distribution is a likely explanation to why cases have been predominantly observed in female moose. In addition, no positivity for prions was detected in any of the lymphoid tissues tested. These characteristics can comply with a prion disease of sporadic appearance [27].

Not all moose were described to have shown clinical signs of disease, and information about clinical appearance prior to death was also missing for some of the cases. However, to assess any possible relationship between pathology, such as degenerative vacuolar change, and expression of clinical signs, fresh non- autolytic material is needed for histopathology. This was not available for these field cases. Moose Sw3 was shot during ordinary hunting and did not show severe clinical signs of CWD, except for the observation that it did not flee when hunters approached her. This animal had the highest amount of PrP^Sc^ as shown by immunohistochemical staining. Conversely, moose Sw2 showed one of the lowest scores for PrP^Sc^ immunohistochemical staining, but instead had showed marked behavioural changes and was emaciated. Therefore, no clear correlation between severity of clinical signs and levels of PrP^Sc^ deposits was found in this study. Moreover, subclinical cases of prion infection are observed in both animals and humans [41], which is in agreement with the finding of CWD in moose No5, shot during hunting and with only discrete observed signs of disease. Additionally, the degree of disease progression in each of the animals was unknown and it was likely different for the different moose. More advanced cases in TSEs show higher levels of PrP^Sc^ accumulation, but may still be at preclinical stages. Taking into account these sources of variation direct comparison between clinical disease or stage of disease progression and PrP^Sc^ accumulation cannot be made.

Susceptibility and disease development in prion diseases are influenced in varying degrees by the genotype of the host, depending on polymorphism in the *PRNP*, the gene encoding the cellular prion protein [42, 43]. In Nordic cervids, reindeer (*Rangifer tarandus*) is the species reported with highest *PRNP* variation followed by red deer (*Cervus elaphus*) and moose (*Alces alces*), while roe deer (*Capreolus capreolus*) has been reported as monomorphic [44, 45]. Similar findings for different deer species have been reported from Britain [46]. In Nordic moose, no association between genotypes and occurrence of CWD has been established and only codon 109 shows variation. Polymorphism at the codon 209 has not been observed in European moose [45]. Most moose were homozygous KK at residue 109 while QQ109 has been reported in three moose [27–44]. Conversely, some genotypes have been found in a larger proportion of the reindeer among the animals infected with CWD [47]. An association between genotype and immunohistochemical profile in the brain has been described in sheep with experimental classical scrapie, seemingly related to the incubation period. Other factors also influence the immunohistochemical profiles, for example the prion strain involved [32]. In this study a clear association between the genotype of the moose and the PrP^Sc^ characteristics could not be established. For example, moose Sw3 had distinct PrP^Sc^ features but had the same genotype as most of the other moose. The two moose with the QQ109 genotype showed some PrP^Sc^ differences as compared with the others, No4 had very low PrP^Sc^ levels and No6 showed high levels of PrP^Sc^ and also a stellate pattern not observed in any of the other moose. There are likely several factors that influence the immunohistochemical profiles besides genotypes, the incubation time in the moose in this study is unknown since they moose were naturally infected.

The findings previously reported in early studies of CWD in moose, such as characteristic intraneuronal PrP^Sc^ deposition and N-terminal truncation [27] were expanded and complemented by this investigation involving more animals. This study identified both common features and also heterogeneity in the PrP^Sc^ distribution in the brain, indicating variation in the presentation of CWD in Nordic moose. The study also corroborated the lack of detection of PrP^Sc^ in lymphoid tissues in all the animals, which is in agreement with the presentation of atypical forms of TSE in bovines and small ruminants. Several transmission studies in murine models are underway, to investigate the types of prion strains in moose with CWD in the Nordic countries. The results of this study help understanding the disease in moose, and might be useful for future diagnosis and identification of various presentations of CWD cases in moose.

### Supplementary Information

**Additional file 1. Example of brain areas sampling.** Here, brain samples from CWD-moose Sw3 were collected to investigate the distribution and abundance of PrP^res^ in the brain by ELISA test. Sample from brainstem area number 14 was not available. Area 16 is normally collected in the primary spoon sample and is therefore added on this picture. For simplification purpose, some of the 16 areas were grouped in the results into seven established brain areas as illustrated with the red boxes. Area 3 and 4 correspond to frontal cortex, area 5, 6 and 10 to temporal and parietal cortex, area 7 to occipital cortex, area 2, 8 and 9 to thalamus, area 11 to midbrain, area 1, 14 and 16 to medulla oblongata and area 12, 13 and 15 to cerebellum.

**Additional file 2. Immunolabelling of PrP**^**Sc**^ **in moose and reindeer controls.** Immunohistochemistry was performed on sections of CWD-positive reindeer brain (**A-B**, level of the obex) and tonsils (**C**), CWD-positive moose brain (**D**, level of the obex), CWD-negative moose (**E**, level of the cerebellum) and reindeer (**F**, level of the obex) with anti-PrP mAbs L42 and SAF84, as indicated. All sections were counterstained with haematoxylin. (Bar = 50 μm).
