# Supplementary figures and images for: Heterogeneity of pathological prion protein accumulation in the brain of moose (Alces alces) from Norway, Sweden and Finland with chronic wasting disease
Source: Vet Res. 2023 Sep 8;54:74. doi: 10.1186/s13567-023-01208-3 (PMC10492377; doi:10.1186/s13567-023-01208-3)

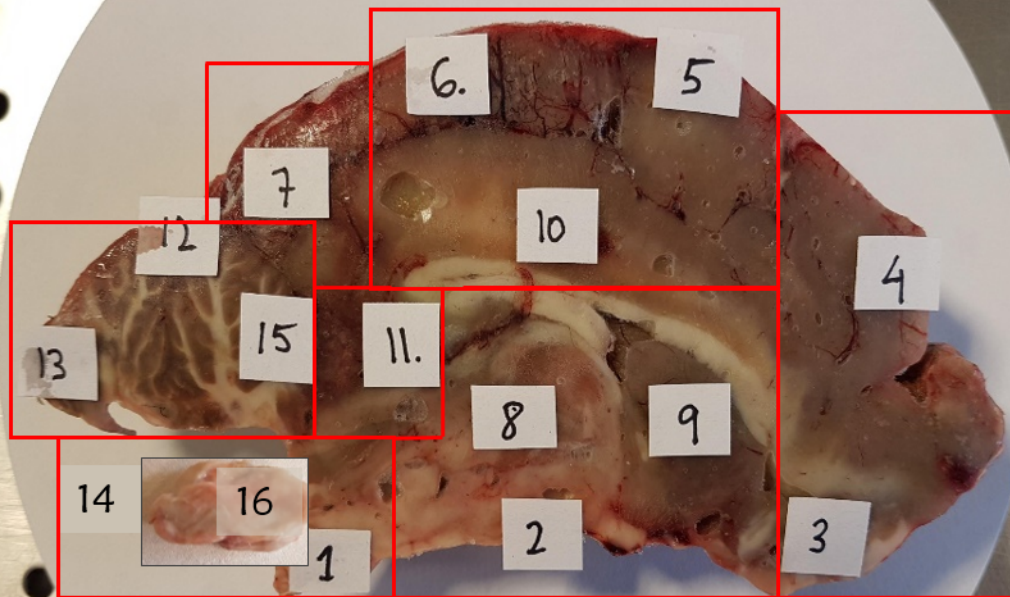

Supplement: Supplementary file 1 — Additional file 1. Example of brain areas sampling. Here, brain samples from CWD-moose Sw3 were collected to investigate the distribution and abundance of PrPres in the brain by ELISA test. Sample from brainstem area number 14 was not available. Area 16 is normally collected in the primary spoon sample and is therefore added on this picture. For simplification purpose, some of the 16 areas were grouped in the results into seven established brain areas as illustrated with the red boxes. Area 3 and 4 correspond to frontal cortex, area 5, 6 and 10 to temporal and parietal cortex, area 7 to occipital cortex, area 2, 8 and 9 to thalamus, area 11 to midbrain, area 1, 14 and 16 to medulla oblongata and area 12, 13 and 15 to cerebellum. [file 13567_2023_1208_MOESM1_ESM.pdf]

L42

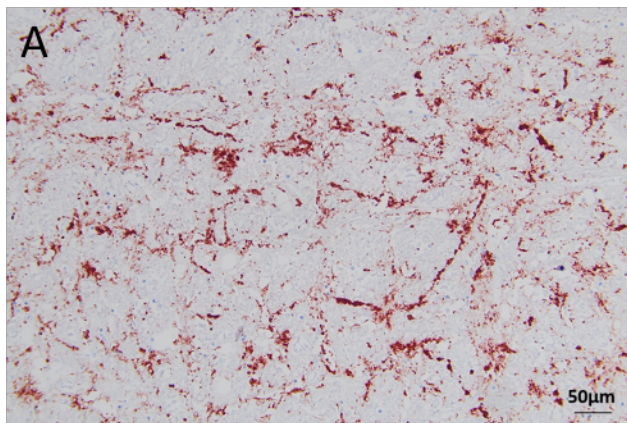

SAF84

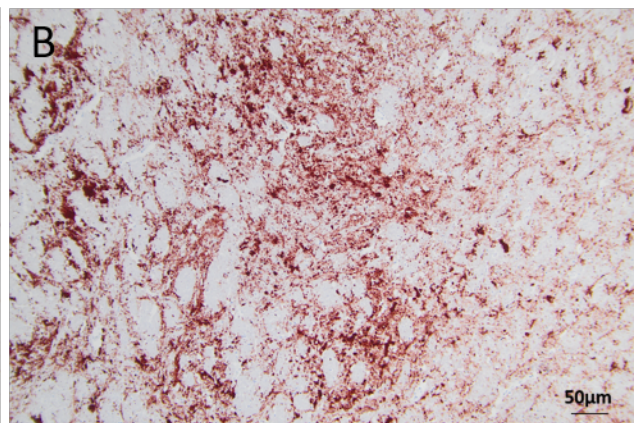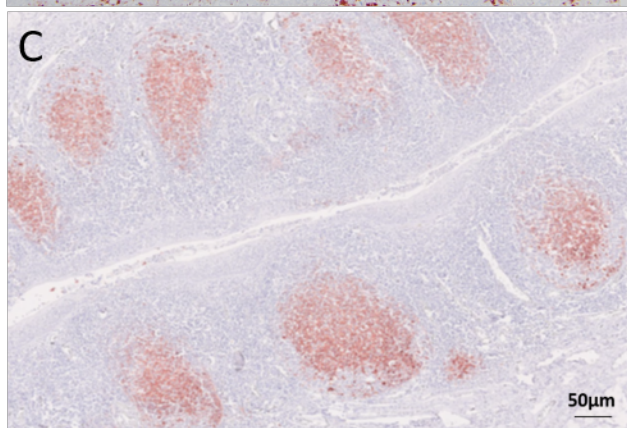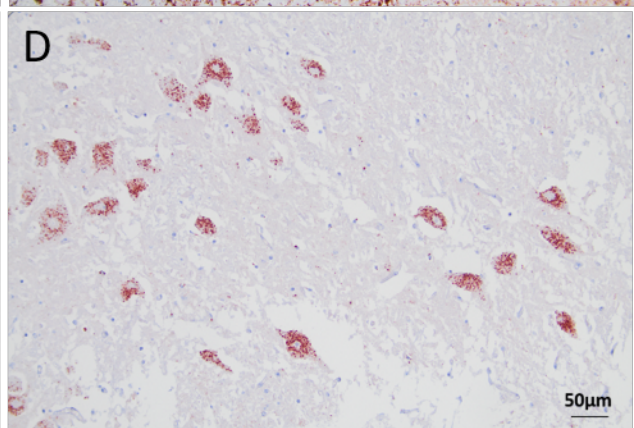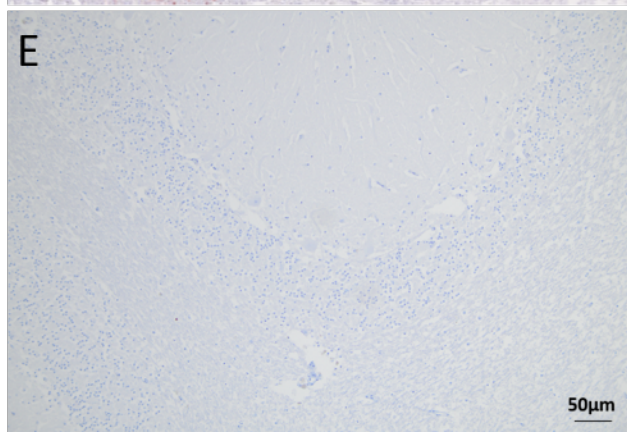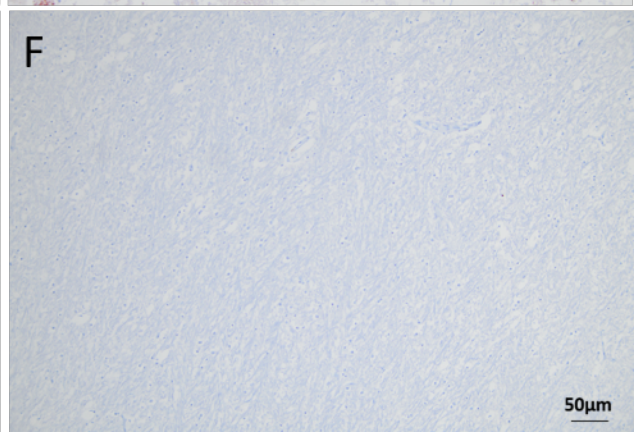

Supplement: Supplementary file 2 — Additional file 2. Immunolabelling of PrPSc in moose and reindeer controls. Immunohistochemistry was performed on sections of CWD-positive reindeer brain (A-B, level of the obex) and tonsils (C), CWD-positive moose brain (D, level of the obex), CWD-negative moose (E, level of the cerebellum) and reindeer (F, level of the obex) with anti-PrP mAbs L42 and SAF84, as indicated. All sections were counterstained with haematoxylin. (Bar = 50 μm). [file 13567_2023_1208_MOESM2_ESM.pdf]
